# Solitary Brain Metastasis From Hepatocellular Carcinoma Masquerading as Acute Ischaemic Stroke: Radiological Diagnosis and Clinical Implications

**DOI:** 10.1155/crra/5706773

**Published:** 2026-07-22

**Authors:** Suzana Lukoo, James Sumawe, Bitesigilwe Mbelwa, Al Karim Kazema, Furaha Malecela, Masanja Singu, Francis Zerd, Jesse Kashabano, Henry Humba, Abel Makubi

**Affiliations:** ^1^ Department of Radiology, Benjamin Mkapa Hospital, Dodoma, Tanzania; ^2^ University of Dar es Salaam, Mbeya College of Health and Allied Sciences, Mbeya, Tanzania, udsm.ac.tz; ^3^ Department of Pathology, Benjamin Mkapa Hospital, Dodoma, Tanzania; ^4^ Department of Oncology, Benjamin Mkapa Hospital, Dodoma, Tanzania; ^5^ Department of Neurosurgery, Benjamin Mkapa Hospital, Dodoma, Tanzania; ^6^ Executive Director, Benjamin Mkapa Hospital, Dodoma, Tanzania

**Keywords:** brain metastasis, computed tomography, diffusion-weighted imaging, hepatocellular carcinoma, magnetic resonance imaging, neurological deficit, stroke mimic

## Abstract

**Background:**

Brain metastases from hepatocellular carcinoma (HCC) are rare, with a reported incidence of approximately 0.2%–2.2% of cases. Although uncommon, they may present with acute focal neurological deficits that closely mimic acute cerebrovascular events, posing significant diagnostic challenges in emergency settings where stroke pathways are routinely prioritised. Failure to recognise an underlying structural lesion in this context may lead to inappropriate management and delayed definitive treatment.

**Case Presentation:**

A 66‐year‐old man with chronic hepatitis B infection and biopsy‐confirmed HCC (Barcelona Clinic Liver Cancer Stage B; serum alpha‐fetoprotein 1000 ng/mL) presented with sudden‐onset right‐sided weakness, expressive aphasia and facial deviation approximately 4 weeks after initial HCC diagnosis. Noncontrast computed tomography (CT) of the brain demonstrated a hypodense intra‐axial lesion in the left frontal lobe with surrounding vasogenic oedema. Because the atypical CT appearance and subsequent heterogeneous contrast enhancement raised suspicion for a neoplastic aetiology, magnetic resonance imaging (MRI) was performed; this revealed a solitary left frontal mass with avid enhancement and facilitated diffusion on diffusion‐weighted imaging, which definitively ruled out acute ischaemic stroke. Gradient echo sequences also demonstrated blooming artefact consistent with intralesional haemorrhage. In the context of known HCC, the imaging findings were consistent with a solitary haemorrhagic brain metastasis presenting as a stroke mimic. The patient was discussed at a multidisciplinary tumour board and subsequently transitioned to palliative care owing to advanced systemic disease and poor functional status.

**Conclusion:**

Solitary brain metastasis from HCC should be considered in the differential diagnosis of patients presenting with acute focal neurological deficits, particularly when initial CT findings are atypical for ischaemic infarction or when a known primary malignancy is present. Early contrast‐enhanced MRI, including diffusion‐weighted sequences, is essential for accurate differentiation of stroke mimics and for guiding timely, appropriate clinical management.

## 1. Introduction

Hepatocellular carcinoma (HCC) is one of the most common primary malignancies worldwide and frequently arises in patients with chronic liver disease, particularly chronic hepatitis B or C infection [1]. Extrahepatic metastases occur in advanced disease and most commonly involve the lungs, lymph nodes, bones and adrenal glands [1, 2]. Brain metastases from HCC are extremely rare, with reported incidence ranging from 0.2% to 2.2% of cases [1, 2]. In sub‐Saharan Africa, where chronic viral hepatitis remains highly prevalent and access to advanced neuroimaging is often limited, recognition of uncommon metastatic patterns is particularly important for timely diagnosis and appropriate referral [3, 4]. Moreover, late‐stage presentation of HCC is common in resource‐constrained settings, potentially increasing the likelihood of haematogenous dissemination to atypical sites including the brain [5].

When brain metastases from HCC occur, they are typically associated with advanced disease and poor prognosis [2, 6]. Neurological manifestations depend on lesion location and may include headache, seizures, altered mental status or focal deficits such as hemiparesis and aphasia [3, 6]. Critically, the onset of neurological symptoms may be abrupt, closely mimicking an acute cerebrovascular event [7, 8]. This clinical overlap represents a significant diagnostic challenge in emergency settings, where stroke pathways are routinely prioritised and where the possibility of an underlying structural lesion may not be considered until imaging reveals atypical features [7]. The stroke mimic presentation is the principal feature that distinguishes this case from routine metastatic disease and justifies its reporting, as it highlights a diagnostic pitfall with direct implications for emergency neuroimaging practice.

Neuroimaging plays a pivotal role in evaluating patients who present with acute neurological deficits. Although noncontrast computed tomography (NCCT) is the first‐line modality in emergency settings due to its availability and speed, it has limited sensitivity in differentiating early metastatic lesions from acute ischaemic infarction, particularly when a mass presents as a hypodense lesion with surrounding oedema [8–10]. Magnetic resonance imaging (MRI) offers superior tissue contrast and, through specific sequences, enables differentiation among the broad differential of ring‐enhancing intracranial lesions—including metastatic disease, pyogenic brain abscess, high‐grade glioma and tumefactive demyelination [8, 11]. Diffusion‐weighted imaging (DWI) is especially critical: Metastatic lesions typically demonstrate facilitated diffusion, whereas pyogenic abscesses exhibit central diffusion restriction reflecting pus viscosity, and acute infarcts show restricted diffusion within a vascular territory [8]. Gradient echo (GRE) and susceptibility‐weighted imaging (SWI) sequences further contribute by detecting intralesional haemorrhage, a frequent feature in hypervascular metastases such as those arising from HCC [6, 11]. In patients with known malignancy who present with acute focal deficits, a systematic MRI protocol incorporating these sequences is therefore essential for accurate diagnosis.

We present a case of solitary brain metastasis from HCC presenting with acute neurological deficits that initially mimicked an acute cerebrovascular event. This report highlights the radiological features that permitted definitive differentiation from stroke, the diagnostic reasoning that guided the transition from CT to MRI and the importance of early cross‐sectional imaging in guiding appropriate clinical management in a sub‐Saharan African institutional setting.

## 2. Case Presentation

A 66‐year‐old male with a background history of chronic hepatitis B infection and recently diagnosed HCC presented to Benjamin Mkapa Hospital, Dodoma, Tanzania, with sudden‐onset right‐sided weakness, expressive aphasia, drooling of saliva and facial deviation. At the time of initial HCC diagnosis 4 weeks prior, the hepatic malignancy had been confirmed by percutaneous liver biopsy and staged as Barcelona Clinic Liver Cancer (BCLC) Stage B (Figure 1). Contrast‐enhanced abdominal CT had demonstrated multifocal heterogeneous hepatic masses with arterial hyperenhancement and portal venous washout (Figure 2). There was no radiological evidence of portal vein thrombosis or extrahepatic metastases. Serum alpha‐fetoprotein (AFP) was markedly elevated at 1000 ng/mL. Liver function assessment demonstrated Child–Pugh Class B with a score of 8.

**Figure 1:**
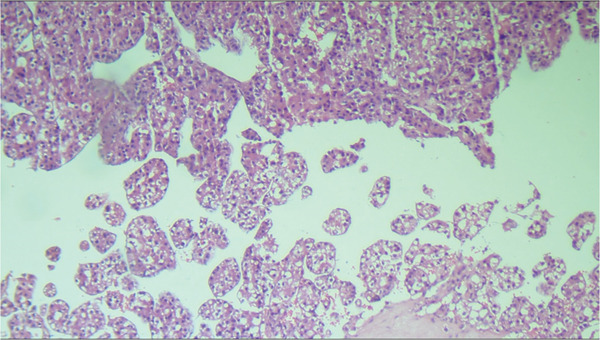
Liver biopsy histopathology (haematoxylin and eosin stain, ×400) showing pleomorphic hepatocytes with granular eosinophilic cytoplasm, prominent nucleoli and increased nuclear‐to‐cytoplasmic ratio, consistent with moderately differentiated hepatocellular carcinoma.

**Figure 2:**
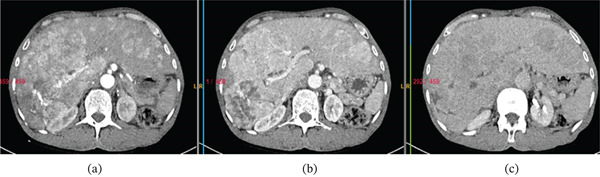
(a–c). Contrast‐enhanced abdominal CT images (arterial and portal venous phases) demonstrating multifocal heterogeneous hepatic masses with arterial hyperenhancement and rapid contrast washout on the portal venous phase, consistent with multifocal hepatocellular carcinoma.

The neurological symptoms developed acutely approximately 4 weeks after the diagnosis of HCC, representing unusually rapid clinical deterioration. This short interval is clinically significant and is suggestive of aggressive tumour biology with possible early haematogenous dissemination from the hypervascular primary tumour [2, 11]. Although no neuroimaging had been performed at the time of initial HCC diagnosis—brain imaging is not routinely included in the staging of asymptomatic patients—the brevity of the interval raises the possibility that metastatic disease was present but clinically occult at baseline.

On clinical examination, the patient was fully conscious with a Glasgow Coma Scale (GCS) score of 15/15. He was haemodynamically stable. Neurological examination revealed expressive aphasia, right‐sided hemiparesis (Medical Research Council Grade 3/5 in the upper and lower limbs) and left lower motor neuron facial nerve involvement, consistent with a left frontal cortical lesion. The remainder of the systemic examination was unremarkable.

Given the acute neurological presentation, an initial working diagnosis of acute cerebrovascular accident was considered. NCCT of the brain demonstrated a hypodense left frontal intra‐axial lesion associated with extensive surrounding vasogenic oedema and mild local mass effect (Figure 3a). Notably, the lesion morphology and oedema pattern were atypical for acute ischaemic infarction: The hypodensity did not conform to a recognised vascular territorial distribution, and the degree of surrounding oedema exceeded what would be expected in the hyperacute phase of stroke. Following intravenous contrast administration, the lesion demonstrated avid heterogeneous enhancement (Figure 3b), raising concern for a neoplastic rather than ischaemic aetiology.

**Figure 3:**
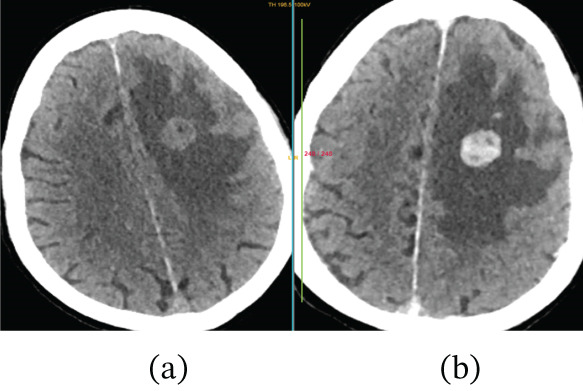
(a–b). Axial brain CT images. (a) Noncontrast CT demonstrates a hypodense intra‐axial lesion in the left frontal lobe with extensive surrounding vasogenic oedema and mild local mass effect; the hypodensity does not conform to a recognised vascular territorial distribution, arguing against acute ischaemic infarction. (b) Contrast‐enhanced CT demonstrates avid heterogeneous enhancement of the lesion, raising concern for a neoplastic aetiology.

In view of the atypical imaging appearance for acute infarction, together with the patient′s known history of HCC, MRI of the brain was subsequently performed for definitive lesion characterisation and exclusion of acute ischaemic stroke. The decision to proceed to MRI was supported by three major considerations: (i) the presence of an enhancing intra‐axial lesion on CT, inconsistent with uncomplicated infarction; (ii) focal cortical neurological deficits not entirely conforming to a single vascular territorial distribution; and (iii) the patient′s high‐risk oncological background, mandating exclusion of intracranial metastatic disease.

MRI of the brain demonstrated a solitary left frontal intra‐axial mass measuring approximately 24 × 20 × 19 mm. On T2‐weighted imaging (T2WI), the lesion appeared heterogeneously hyperintense with extensive surrounding vasogenic oedema extending into the adjacent white matter and causing mild local mass effect without significant midline shift (Figure 4a). Postcontrast T1‐weighted imaging (T1WI) revealed avid enhancement throughout the lesion volume (Figure 4b). This solid, avid enhancement pattern, in conjunction with the known systemic malignancy, strongly favoured metastatic disease over a pyogenic abscess, which characteristically demonstrates a nonenhancing cystic cavity with a thin, smooth, peripheral rim [4, 12]. Dedicated SWI was not available at our facility; therefore, GRE sequences were used to assess for haemorrhage, demonstrating prominent blooming artefact within the lesion consistent with intralesional bleeding—a recognised feature of hypervascular metastases from HCC [7]. DWI and apparent diffusion coefficient (ADC) mapping demonstrated facilitated diffusion within the lesion, a finding that argues against both acute ischaemic infarction (which characteristically restricts diffusion) and pyogenic abscess (which typically demonstrates central diffusion restriction reflecting pus viscosity) [4]. No additional intracranial lesions were identified.

**Figure 4:**
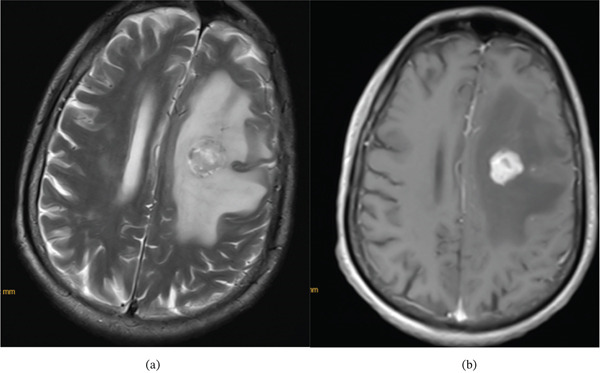
(a–b). Brain MRI. (a) Axial T2‐weighted image (T2WI) demonstrating a left frontal hyperintense intra‐axial lesion with a heterogeneous signal, extensive surrounding vasogenic oedema extending into the adjacent white matter and associated mass effect without significant midline shift. (b) Postcontrast axial T1‐weighted image (T1WI) showing avid enhancement.

In the context of a hypervascular tumour such as HCC, early brain metastases may remain asymptomatic until rapid tumour growth, perilesional oedema or haemorrhagic transformation leads to clinical presentation [7, 11]. The short interval between HCC diagnosis and neurological deterioration in this case is consistent with this proposed mechanism.

Based on the imaging findings—avid enhancement, intralesional haemorrhage and facilitated diffusion—together with the clinical context of biopsy‐proven HCC, a diagnosis of solitary haemorrhagic brain metastasis from HCC was established. The lesion had presented clinically as a stroke mimic.

The case was discussed at a multidisciplinary tumour board comprising representatives from radiology, oncology, neurosurgery and pathology. Given the advanced systemic disease (BCLC Stage B, Child–Pugh B), poor oncological prognosis and the patient′s functional status, consensus was reached that neurosurgical intervention and radiotherapy were not indicated. Management was directed towards palliative care and symptomatic control.

## 3. Discussion

This case illustrates a rare but clinically significant presentation: solitary brain metastasis from HCC manifesting as an acute stroke mimic in a patient with recently diagnosed disease. Brain metastases from HCC are uncommon, with reported incidence ranging from 0.2% to 2.2%, making the brain a rare site of metastatic spread compared with the lungs, lymph nodes and bones [1, 2]. Although brain metastases collectively represent the most common intracranial tumours in adults—reflecting increasing cancer survival and improved imaging detection—those arising from HCC are disproportionately rare and carry a particularly poor prognosis, with median survival generally less than 6 months [2, 5, 7, 13].

From a pathophysiological perspective, intracranial metastasis from HCC occurs via haematogenous dissemination, facilitated by the highly vascular nature of the primary tumour [6, 11]. The frontal and parietal lobes are preferentially involved, reflecting regional cerebral blood flow distribution [6]. In the present case, the left frontal location is consistent with this pattern. The unusually short interval of 4 weeks between HCC diagnosis and neurological presentation is noteworthy and suggests aggressive tumour biology with early haematogenous seeding. The markedly elevated AFP (1000 ng/mL) and BCLC Stage B classification further support a biologically aggressive phenotype [2, 7].

A central diagnostic challenge in this case was the broad differential diagnosis of an avidly enhancing intracranial lesion with surrounding vasogenic oedema in a 66‐year‐old immunocompetent patient. This radiological pattern is nonspecific and encompasses several distinct pathological entities. Metastatic disease from a known extracranial primary malignancy is the most likely diagnosis in this clinical context, but alternative possibilities must be systematically considered [9, 12]. Pyogenic brain abscess typically produces a thin, smooth‐walled peripheral enhancing rim surrounding a central nonenhancing purulent core; this stands in sharp contrast to the solid, avidly enhancing observed throughout the mass in our patient. Additionally, abscesses present with central diffusion restriction on DWI reflecting pus viscosity—a feature absent in this case, where facilitated diffusion was observed [9]. High‐grade glioma, particularly glioblastoma multiforme, produces ring‐enhancement, more infiltrative, irregular margins with involvement of white matter tracts and crossing of the corpus callosum [9, 12]. Tumefactive demyelination was considered unlikely given the absence of clinical or radiological features of demyelinating disease. In the Tanzanian context, regionally relevant infectious aetiologies—including tuberculoma and neurotoxoplasmosis—warranted consideration but were judged unlikely given the patient′s immunocompetent status, the enhancement morphology and the absence of systemic features of infection [12]. The diagnosis of metastasis was therefore favoured on the basis of the composite imaging characteristics, the known systemic malignancy, the facilitated diffusion pattern and the absence of clinical features supporting alternative diagnoses.

The role of advanced MRI techniques in differentiating intracranial lesions deserves particular emphasis in this case. DWI is arguably the most critical sequence in the emergency evaluation of acute neurological deficits, as it simultaneously excludes acute infarction (which restricts diffusion within a vascular territory) and supports the diagnosis of metastasis (which characteristically demonstrates facilitated diffusion) [9]. In the present case, the facilitated diffusion on DWI and ADC mapping was a key factor in redirecting the diagnosis away from stroke. GRE sequences provided additional diagnostic value by demonstrating blooming artefact consistent with intralesional haemorrhage, a finding of particular relevance in HCC metastases given the hypervascular nature of the primary tumour [7, 12]. Although current literature highlights SWI as an optimal tool for detecting microhaemorrhage within metastatic lesions, dedicated SWI was not available in our clinical setting, making standard GRE sequences the essential alternative to successfully identify the intralesional haemorrhage [12].

The mechanism of acute neurological deterioration in metastatic brain disease is multifactorial and warrants discussion, as the specific mechanism has implications for both acute management and prognostic counselling [4, 5]. In this case, several mechanisms may have contributed. Acute perilesional vasogenic oedema, resulting from disruption of the blood–brain barrier by tumour infiltration, can produce rapid neuronal dysfunction and focal deficits that evolve over hours [7, 11]. Intratumoral haemorrhage, a well‐recognised feature of HCC brain metastases owing to the hypervascular nature of the primary tumour, may also produce sudden neurological decline and can closely mimic haemorrhagic stroke on initial CT [7]. The GRE blooming artefact observed in this case supports intralesional haemorrhage as a contributing factor. Vascular compromise from local mass effect on adjacent cortical vessels represents a third mechanism. These pathways are not mutually exclusive: In a hypervascular metastasis with surrounding oedema, a combination of haemorrhage and oedema‐mediated neuronal dysfunction likely accounts for the abrupt onset observed [5, 11].

Management of brain metastases from HCC is guided by systemic disease burden, hepatic functional reserve, intracranial disease characteristics and performance status [4, 5]. Standard treatment options in well‐resourced settings include surgical resection for accessible solitary lesions, stereotactic radiosurgery (SRS), whole‐brain radiotherapy (WBRT) and systemic therapy including targeted agents and immunotherapy [4, 13]. However, in resource‐limited settings such as our institution in Dodoma, Tanzania, access to SRS is not currently available, and WBRT capacity is constrained. These infrastructural realities directly influenced the management of this patient.

Care was therefore directed towards symptom control and supportive management. This included corticosteroid therapy for reduction of perilesional oedema, analgesic optimisation, regular neurological monitoring and comprehensive family counselling regarding prognosis and expected disease progression. The median survival of HCC brain metastases ranges from 1 to 6 months following diagnosis [2, 7], and this was communicated to the patient′s relatives as part of shared decision‐making. In a resource‐limited context, the ethical imperative to provide honest prognostic information, to avoid futile interventions and to optimise quality of life takes on heightened practical significance [4].

Several limitations of this case must be acknowledged. The principal limitation is the absence of histopathological confirmation of the intracranial lesion. The diagnosis of brain metastasis rests entirely on imaging characteristics in the clinical context of biopsy‐proven HCC. Although this clinicoradiological approach is widely accepted in clinical practice—particularly in patients with advanced systemic disease, high surgical risk and poor performance status—it introduces diagnostic uncertainty, and the possibility of a concurrent primary intracranial pathology, however unlikely, cannot be completely excluded [4].

Despite these limitations, this case provides important clinical and radiological lessons. It demonstrates that brain metastasis from HCC, though rare, can present as a convincing stroke mimic and underscores the importance of maintaining a broad differential diagnosis for enhancing intracranial lesions presenting with acute focal neurological deficits. It further highlights the critical role of systematic MRI evaluation—incorporating DWI, contrast‐enhanced sequences and GRE imaging—in distinguishing metastatic disease from acute infarction, abscess and primary brain tumours in emergency settings.

## 4. Conclusion

Solitary brain metastasis from HCC, though rare, can present with acute focal neurological deficits that convincingly mimic stroke, as illustrated by this case. The abrupt onset of symptoms combined with nonspecific CT findings in the emergency setting created a diagnostic challenge that was resolved only through systematic contrast‐enhanced MRI evaluation. Three key clinical lessons emerge. First, in patients with known or recently diagnosed HCC who develop acute neurological deficits, brain metastasis should be included in the differential even when the presentation strongly suggests a cerebrovascular event. Second, early MRI incorporating DWI and GRE sequences is indispensable for differentiating stroke mimics from genuine infarction, particularly when CT findings are atypical or when an oncological history is present. Third, in resource‐limited settings where access to SRS and advanced systemic therapies may be restricted, timely radiological diagnosis remains essential to guide realistic management planning and to facilitate honest, evidence‐based prognostic counselling for patients and families.

A key limitation of this report remains the absence of histopathological confirmation of the intracranial lesion. This underscores the importance of careful clinicoradiological correlation when managing advanced cancer patients, and highlights the ongoing need for structured imaging protocols in emergency neurological presentations associated with known malignancy.

## Author Contributions

Suzana Lukoo: conceptualisation, investigation, writing—original draft, writing—review and editing. James Sumawe: conceptualisation, formal analysis, investigation, methodology, writing—original draft, writing—review and editing. Bitesigilwe Mbelwa: investigation, writing—review and editing. Al Karim Kazema: investigation, writing—review and editing. Furaha Malecela: writing—review and editing. Masanja Singu: writing—review and editing. Francis Zerd: writing—review and editing. Jesse Kashabano: writing—review and editing. Henry Humba: writing—review and editing. Abel Makubi: supervision, writing—review and editing.

## Funding

No funding was received for this manuscript.

## Consent

Written informed consent was obtained from the patient′s next of kin for the publication of this case report and accompanying images.

## Conflicts of Interest

The authors declare no conflicts of interest.

## Data Availability

The data that support the findings of this study are available from the corresponding author upon reasonable request.
